# The Metropolitan Street Ambulance Association

**Published:** 1904-02-27

**Authors:** Arthur James

**Affiliations:** Honorary Secretary


### Editor's Letter-Box.

**[Our Correspondents are reminded that prolixity is a great bar to publication, and that brevity of style and conciseness of statement greatly facilitate early insertion.]**

### THE METROPOLITAN STREET AMBULANCE ASSOCIATION.

*To the Editor of* "The Hospital."

Sir,—In view of the approaching London County Council elections, may I be allowed to call attention to the purpose of the Metropolitan Street Ambulance Association, of which Mr. Reginald Harrison, F.R.C.S., is President.

At present this Association is composed entirely of London medical men, and is thoroughly representative of the whole profession.

The members include the President of the Royal College of Physicians, the President of the Royal College of Surgeons, members of the staffs of all the London hospitals, and the best-known men from every County Council constituency.

The intention of the Association is to impress on the public and their representatives in the County Council the urgent need of an efficient Street Ambulance organisation for London, and to collect such irrefutable evidence from every metropolitan district as shall lead Parliament to sanction the establishment of such a service by the Council.

In order to carry out these objects the executive committee have appointed one or more medical men in each of the 58 constituencies to act as local secretaries, and, when necessary, to become the chairmen of sub-committees.

The reports received from every quarter prove beyond doubt that the public generally are keenly interested in furthering the adoption of a scheme of this nature, and it is expected that candidates of all parties will therefore give it their active support.

London has now at its disposal the following arrangements for dealing with the 12,000 to 15,000 street casualties requiring removal each year.

1. Mr. H. L. Bischoffsheim has placed in various parts of London, at his own expense, 56 ambulances to be used by anyone who will fetch them.

2. The St. John Ambulance Association has about 25 stations where stretchers or litters are available for anyone who knows where to find them, besides three stations where officials are on duty.

3. The Police keep at each of their stations one or more ambulances or litters, often of a very unsatisfactory type, to be used by themselves for drunken people or for casualties. They also have three horse ambulances for the whole of London, but these are not available at a moment's notice.

With such arrangements, not even aided by telephonic communication (except in the case of the chief station of the St. John Association), it is not surprising to learn that from 8,000 to 10,000 of these severe accident cases have to be taken to hospitals or to their own homes each year in cabs or in other unsuitable conveyances.

Only medical men can thoroughly appreciate the dreadful waste of life, added suffering, and further injury that must take place under circumstances of this kind.

Those who are accustomed to see the dangerous accidents occurring daily in the neighbourhood of the London Docks and elsewhere, and the manner in which they arrive at the hospitals, are astonished at the continued delay of the authorities in providing the necessary ambulances.

On investigation it would probably be found that the expense of an adequate scheme would not be so great as is often supposed, as many of the existing arrangements might advantageously be retained or adapted: the police might continue their excellent "First Aid" work, but could be relieved of the necessity of accompanying their patients for long distances. The telephones belonging to the Post Office and to private individuals might by arrangement be made available, and ample room could be left in any scheme for such philanthropic work as has been so generously done by Mr. H. L. Bischoffsheim and by the St. John Ambulance Association.

I am, your obedient servant,
Arthur James, M.D., D.P.H.,
Honorary Secretary.

